# Primary small cell-like hepatocellular carcinoma arising in a patient with fatty liver disease without cirrhosis: a case report and literature review

**DOI:** 10.1093/gastro/goaf061

**Published:** 2025-07-19

**Authors:** Hyun Bin Choi, Jeong-Ju Yoo, Susie Chin, Sang Gyune Kim, Young-Seok Kim

**Affiliations:** Department of Internal Medicine, Soonchunhyang University Bucheon Hospital, Bucheon, Republic of Korea; Division of Gastroenterology and Hepatology, Department of Internal Medicine, Soonchunhyang University Bucheon Hospital, Bucheon, Republic of Korea; Department of Pathology, Soonchunhyang University Bucheon Hospital, Bucheon, Republic of Korea; Division of Gastroenterology and Hepatology, Department of Internal Medicine, Soonchunhyang University Bucheon Hospital, Bucheon, Republic of Korea; Division of Gastroenterology and Hepatology, Department of Internal Medicine, Soonchunhyang University Bucheon Hospital, Bucheon, Republic of Korea

## Introduction

Hepatocellular carcinoma (HCC) is the sixth-most common cancer globally and the third-leading cause of cancer-related mortality [1]. Its incidence is continuing to rise, driven by the growing prevalence of metabolic dysfunction-associated steatotic liver disease [2]. In the 2019 WHO classification (5th edition), HCC was categorized into eight subtypes based on molecular and histopathological characteristics [3]. However, small cell-like HCC is extremely rare and was not included in this classification. Here, we present the first comprehensive case of small cell-like HCC.

## Case report

A 74-year-old South Korean male presented to a local clinic with persistent fever of 38°C for 15 days. Abdominal ultrasound revealed multiple masses in the left hepatic lobe, leading to his referral to our hospital. His past medical history included hypertension, diabetes mellitus, hyperlipidemia, and prior robotic-assisted radical prostatectomy for prostate cancer. He had no significant liver-related history except for hepatic hemangioma and severe fatty liver. His fibrosis 4 score was 3.09, indicating an intermediate risk of liver fibrosis. He was a social drinker.

Upon admission, his vital signs were stable and physical examination was unremarkable. He had a height of 1.596 m, a weight of 71.3 kg, and a body mass index of 27.99 kg/m^2^. Blood test results were as follows: white blood cell count, 10.77 × 10³/µL; hemoglobin, 107 g/L; platelets, 454 × 10³/µL; prothrombin time/international normalized ratio, 1.15; total bilirubin, 14.36 µmol/L; albumin, 40 g/L; creatinine, 88.4 µmol/L. Hepatitis B surface antibody and hepatitis C antibody were negative, while hepatitis B antibody was positive. The alpha-fetoprotein (AFP) level was elevated to 33,694.0 ng/mL and the protein induced by vitamin K absence or antagonist-II (PIVKA-II) was elevated to 3,475.0 mAU/mL. The carbohydrate antigen 19-9 level was within normal limits, at 10.6 kU/L.

**Figure 1.**
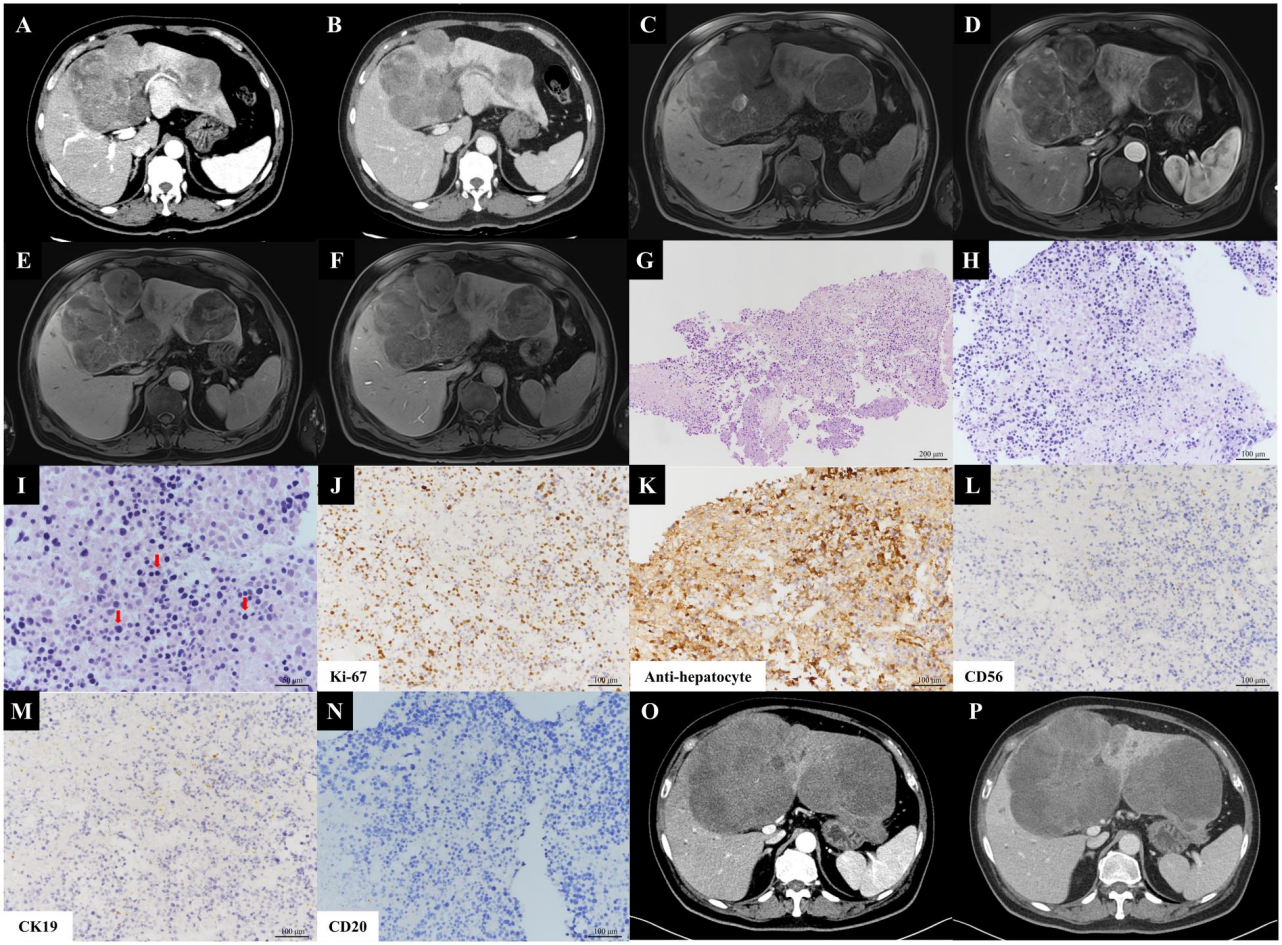
Pre- and post-treatment liver dynamic CT, MRI, and liver biopsy. (A) Pretreatment liver dynamic CT. Arterial phase enhancement. (B) Portal phase washout. Multiple lobulated masses and direct invasion of the left umbilical portal vein and tumor thrombosis. (C) Pretreatment liver dynamic MRI. Pre-contrast. (D) Arterial phase. (E) Delayed phase. (F) Hepatobiliary phase. Findings consistent with liver dynamic CT. (G) Liver biopsy, H&E staining, ×100; (H) ×200; (I) ×400. Poorly differentiated carcinoma with nearly total necrosis with some viable small round cells observed. (J) Liver biopsy, immunohistochemical staining. Ki-67 stain, ×200. High Ki-67 proliferative index. (K) Anti-hepatocyte stain, ×200. Focally positive. (L) CD56 staining, ×200. Negative. (M) CK19 staining, ×200. Negative. (N) CD20 staining, ×200. Negative. (O) Post-treatment liver dynamic CT. Arterial phase enhancement. (P) Portal phase washout. Significant progression of extensive HCC lesions and increased extent of tumor thrombosis in the left portal vein compared with pretreatment liver dynamic CT. CT = computed tomography, MRI = magnetic resonance imaging, H&E = hematoxylin and eosin; HCC = hepatocellular carcinoma.

Three-phase liver dynamic computed tomography revealed multiple lobulated masses involving segments S3, S4, and S5, with the largest measuring 12 cm. These masses showed arterial phase enhancement and portal phase washout. Findings suggested invasion of the left umbilical portal vein and tumor thrombosis. Previous hepatic hemangiomas remained stable (Figure 1A and B). Liver dynamic magnetic resonance imaging confirmed these findings (Figure 1C–F).

Percutaneous liver biopsy revealed poorly differentiated carcinoma with extensive necrosis and viable small round cells (Figure 1G–I). The immunohistochemistry demonstrated a high Ki-67 proliferative index. Markers for chromogranin, CD56, CK19, and CD20 were negative, excluding neuroendocrine tumors, cholangiocarcinoma, and lymphoma. Focal positivity for anti-hepatocyte antibody supported a hepatocytic origin (Figure 1J–N). Consequently, the patient was diagnosed with small cell-like HCC, classified as Barcelona Clinic Liver Cancer stage C and modified Union for International Cancer Control stage IVA. He had an Eastern Cooperative Oncology Group performance status of 0, Child–Pugh class A (score of 5), and a Model for End-stage Liver Disease score of 8. We initiated systemic therapy with atezolizumab/bevacizumab and planned radiation therapy targeting the portal vein tumor thrombosis [4, 5].

The patient completed the first cycle of atezolizumab/bevacizumab chemotherapy without acute side effects. Upon returning for the second cycle, his AFP and PIVKA-II had markedly increased to 74,770.0 ng/mL and 15,714.4 mAU/mL, respectively. One week after the second cycle, he was readmitted for radiotherapy targeting the left portal vein tumor thrombosis and the segment S4 lesion, followed by the third cycle of chemotherapy. Tumor markers continued to escalate (AFP, 131,759.0 ng/mL; PIVKA-II, 16,803.9 mAU/mL). After the third cycle, AFP rose to 196,312.0 ng/mL and PIVKA-II to 24,555.5 mAU/mL (Supplementary Figure S1). Follow-up liver dynamic computed tomography showed disease progression with enlarging liver lesions and worsening portal vein tumor thrombosis (Figure 1O and P). Second-line systemic therapy was considered, but the patient declined further treatment.

## Discussion

The terms “small cell-like hepatocellular carcinoma” and “small cell carcinoma of the liver” may be confusing due to their similar names. “Small cell-like hepatocellular carcinoma” is a histologic variant of HCC originating from hepatocytes, whereas “small cell carcinoma of the liver,” also referred to as “hepatic small cell carcinoma,” is a type of neuroendocrine carcinoma arising from neuroendocrine cells [6]. Although “small cell carcinoma of liver” has been reported in ∼30 cases, this is the first documented case of “small cell-like hepatocellular carcinoma.”

In the 2019 WHO classification, ∼65% of HCCs lacking distinct histologic features are classified as not otherwise specified HCC (NOS-HCC). The remaining 35% are divided into eight subtypes: steatohepatitic, clear cell, macrotrabecular-massive, scirrhous, chromophobe, fibrolamellar, neutrophil-rich, and lymphocyte-rich [8]. A summary of the features for each subtype is provided in Supplementary Table S1 [3, 7, 8]. Small cell-like HCC has not been categorized due to its rarity. A literature search revealed only a poster by Fitzpatrick *et al.* [9] suggesting similar morphology.

This patient had no significant liver-related history except for severe fatty liver. A rapidly growing cluster of multiple liver masses was detected on magnetic resonance imaging performed 17 months after the previous scan. Based on histological and immunohistochemical findings, the diagnosis of HCC with small cell morphology was confirmed. Given the atypical nature of this HCC subtype, a multidisciplinary team approach was implemented to discuss and plan management [10].

In this case, HCC developed directly from fatty liver without progressing through liver cirrhosis. Although the direct role of small cell histology in this progression is uncertain, its highly aggressive and rapidly growing nature may have significantly contributed to the accelerated course of the disease. Considering the aggressive behavior of small cell-like HCC, careful surveillance is warranted in patients with fatty liver.

Image findings of small cell-like HCC were indistinguishable from conventional HCC. Therefore, conducting a biopsy promptly after imaging appears to be essential for the accurate and timely diagnosis and treatment of small cell-like HCC.

Despite atezolizumab/bevacizumab therapy, the patient’s disease progressed rapidly. This suggests the limited responsiveness of small cell-like HCC to current first-line therapy for conventional HCC. As a result, second-line therapy with lenvatinib was recommended, but was not administered due to the patient’s refusal. Long-term monitoring and follow-up will be crucial to assess the therapeutic efficacy of alternative treatments for managing this rare and aggressive variant of HCC.

## Supplementary Material

goaf061_Supplementary_Data
