# Supplementary material for: Primary small cell-like hepatocellular carcinoma arising in a patient with fatty liver disease without cirrhosis: a case report and literature review
Source: Gastroenterol Rep (Oxf). 2025 Jul 19;13:goaf061. doi: 10.1093/gastro/goaf061 (PMC12275463; doi:10.1093/gastro/goaf061)
Supplement: goaf061_Supplementary_Data [file goaf061_supplementary_data.zip › Supplementary material.docx]

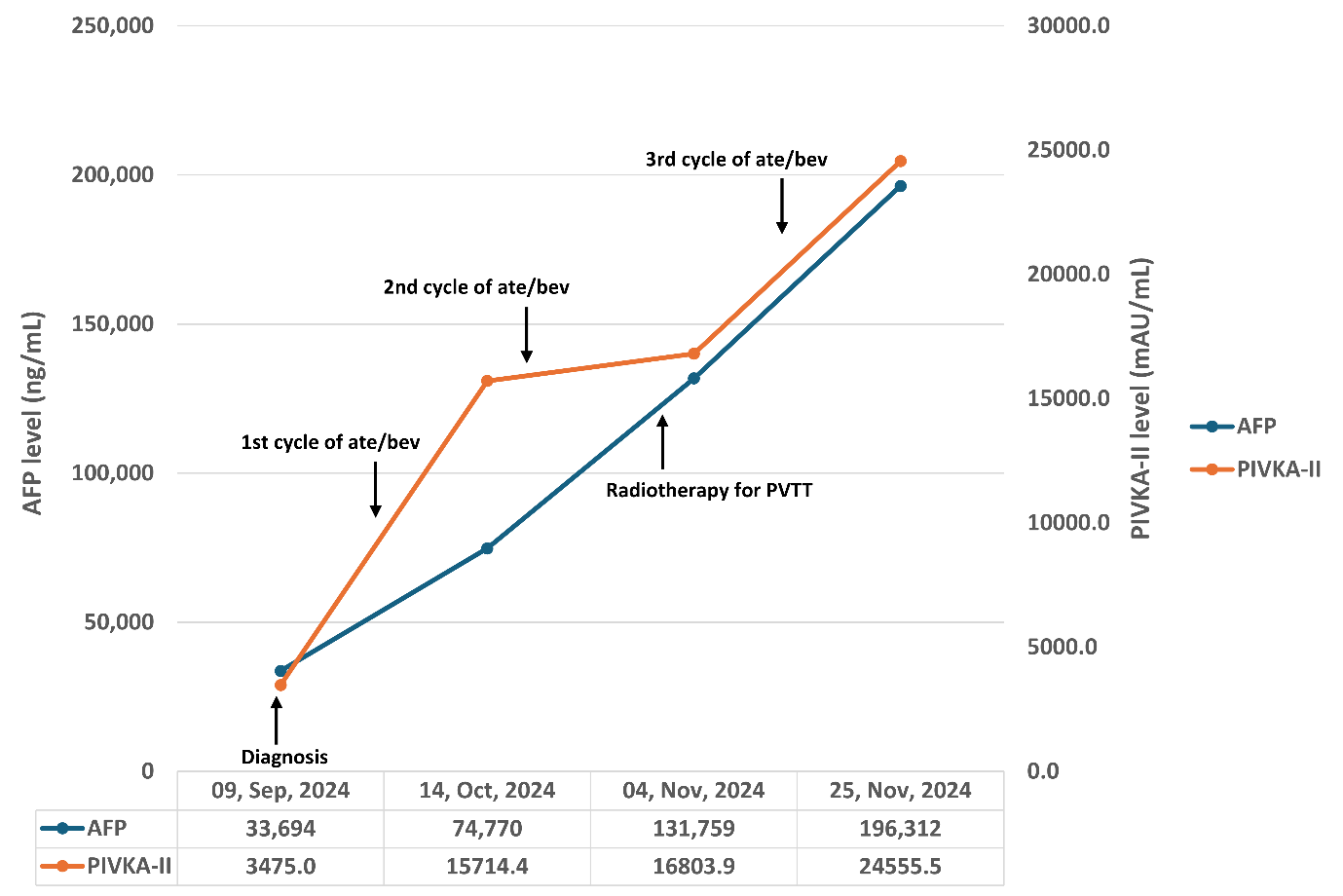


**Supplementary Figure S1.** AFP and PIVKA-II level progression during treatment.

Abbreviations: AFP, alpha-fetoprotein; ate/bev, atezolizumab/bevacizumab; PIVKA-II, protein induced by vitamin K absence or antagonist-II; PVTT, portal vein tumor thrombosis

**Supplementary Table S1. Histopathologic subtypes of hepatocellular carcinoma according to 2019 World Health Organization classification of digestive system tumors (5th edition)**

| **Subtype** | **Frequency among all HCCs (%)** | **Histopathologic features** | **Clinical features** | **Prognosis*** |
| --- | --- | --- | --- | --- |
| **Steatohepatitic** | 5–20 | Steatohepatitis: cell ballooning, inflammation, pericellular fibrosis, Mallory-Denk bodies | MASLD, MASH | Similar |
| **Clear cell** | 3–7 | Cytoplasmic accumulation of glycogen, clear cell morphology | Important to differentiate from metastatic clear cell RCC | Better |
| **MTM** | 5 | Macrotrabecular architecture with satellite nodules and vascular invasion | Hepatitis B, high serum AFP level | Worse |
| **Scirrhous** | 4 | Dense fibrous stroma | Weaker association with hepatitis B, lower serum AFP level | Unclear |
| **Chromophobe** | 3 | Light cytoplasm, abrupt focal nuclear anaplasia, pseudocysts | Chronic hepatitis B | Similar |
| **Fibrolamellar** | 1 | Well-differentiated neoplastic cells with deeply eosinophilic and granular cytoplasm, dense fibrous lamellae | Young age, no background liver disease | Similar in non-cirrhotic livers, better in cirrhotic livers |
| **Neutrophil-rich** | <1 | Marked infiltrates by neutrophils | Older age, elevation in WBC count, serum IL-6, CRP | Worse |
| **Lymphocyte-rich** | <1 | Prominent lymphocytic infiltrate, predominance of cytotoxic CD8^+^ lymphocytes | Not related to EBV | Better |

*Prognosis compared to conventional HCC

Abbreviations: AFP, alpha-fetoprotein; CRP, C-reactive protein; EBV, Epstein-Barr virus; HCC, hepatocellular carcinoma; IL-6, interleukin-6; MASH, metabolic dysfunction-associated steatohepatitis; MASLD, metabolic dysfunction-associated steatotic liver disease; MTM, macrotrabecular-massive; RCC, renal cell carcinoma; WBC, white blood cell.
