# Supplementary figures and images for: Primary small cell-like hepatocellular carcinoma arising in a patient with fatty liver disease without cirrhosis: a case report and literature review
Source: Gastroenterol Rep (Oxf). 2025 Jul 19;13:goaf061. doi: 10.1093/gastro/goaf061 (PMC12275463; doi:10.1093/gastro/goaf061)

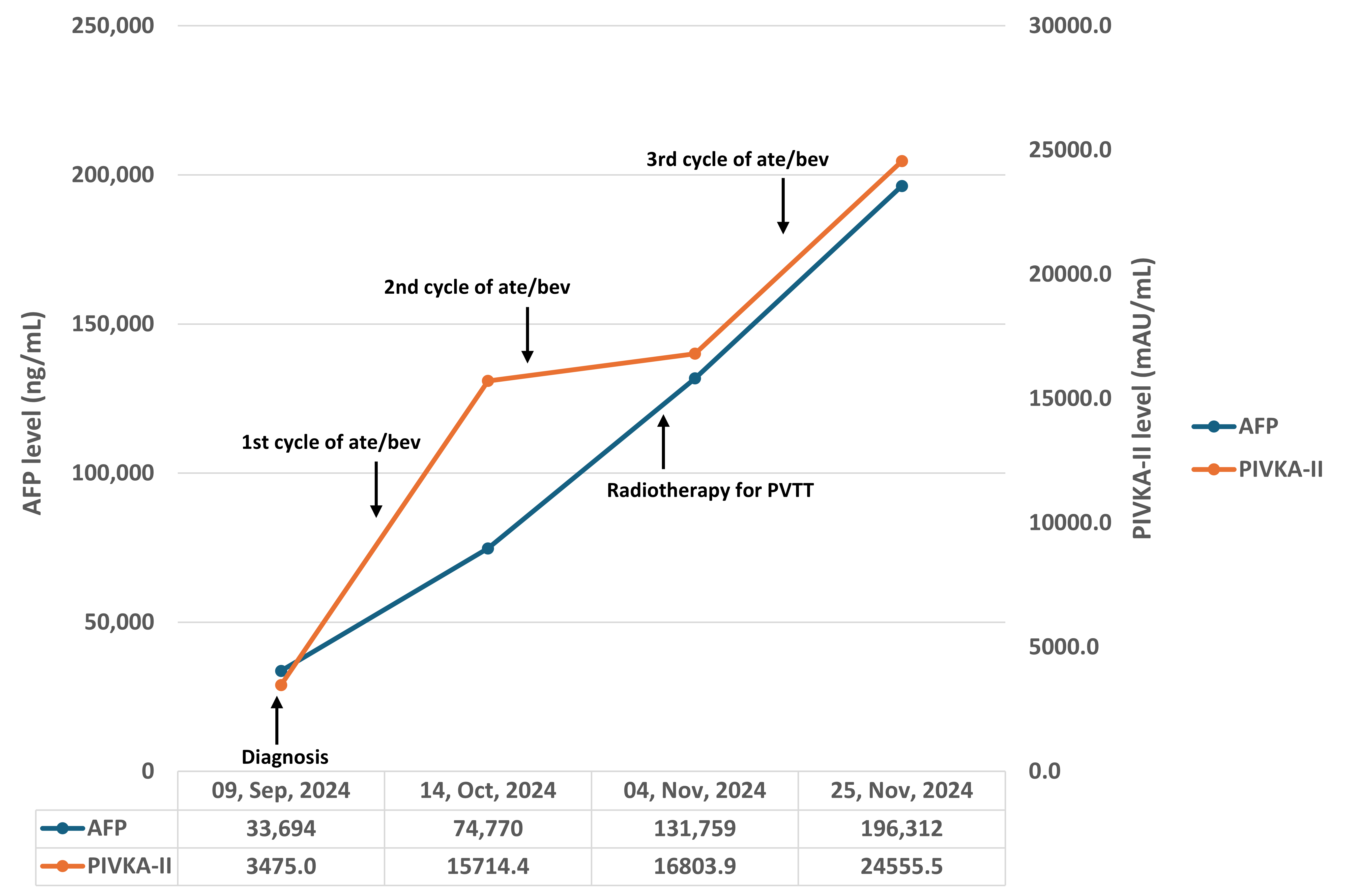

Supplement: goaf061_Supplementary_Data [file goaf061_supplementary_data.zip › Supplementary Figure S1.tif]
